# Identification and characterization of nuclear and nucleolar localization signals in 58-kDa microspherule protein (MSP58)

**DOI:** 10.1186/s12929-015-0136-0

**Published:** 2015-05-16

**Authors:** Chuan-Pin Yang, Chi-Wu Chiang, Chang-Han Chen, Yi-Chao Lee, Mei-Hsiang Wu, Yi-Huan Tsou, Yu-San Yang, Wen-Chang Chang, Ding-Yen Lin

**Affiliations:** Institute of Bioinformatics and Biosignal Transduction, College of Bioscience and Biotechnology, National Cheng Kung University, Tainan, 70101 Taiwan ROC; Department of Pharmacology, College of Medicine, National Cheng Kung University, Tainan, 70101 Taiwan ROC; Institute of Molecular Medicine, College of Medicine, National Cheng Kung University, Tainan, 70101 Taiwan ROC; Infectious Diseases and Signaling Research Center, National Cheng Kung University, Tainan, 70101 Taiwan ROC; Institute for Cancer Biology and Drug Discovery, College of Medical Science and Technology, Taipei Medical University, Taipei, 11031 Taiwan ROC; Graduate Institute of Medical Sciences, College of Medicine, Taipei Medical University, Taipei, 11031 Taiwan ROC; Center for Neurotrauma and Neuroregeneration, Taipei Medical University, Taipei, 11031 Taiwan ROC; Program for Neural Regenerative Medicine, College of Medical Science and Technology, Taipei Medical University, Taipei, 11031 Taiwan ROC; Center for Translational Research in Biomedical Sciences, Kaohsiung Chang Gung Memorial Hospital, Kaohsiung, 83301 Taiwan ROC; Department of Applied Chemistry, National Chi Nan University, Puli, Nantou, 54561 Taiwan ROC

**Keywords:** 58-kDa Microspherule Protein, Nucleolus, Nuclear localization signal, Nucleolar localization signal, Importins

## Abstract

**Background:**

MSP58 is a nucleolar protein associated with rRNA transcription and cell proliferation. Its mechanism of translocation into the nucleus or the nucleolus, however, is not entirely known. In order to address this lack, the present study aims to determine a crucial part of this mechanism: the nuclear localization signal (NLS) and the nucleolar localization signal (NoLS) associated with the MSP58 protein.

**Results:**

We have identified and characterized two NLSs in MSP58. The first is located between residues 32 and 56 (NLS1) and constitutes three clusters of basic amino acids (KRASSQALGTIPKRRSSSRFIKRKK); the second is situated between residues 113 and 123 (NLS2) and harbors a monopartite signal (PGLTKRVKKSK). Both NLS1 and NLS2 are highly conserved among different vertebrate species. Notably, one bipartite motif within the NLS1 (residues 44–56) appears to be absolutely necessary for MSP58 nucleolar localization. By yeast two-hybrid, pull-down, and coimmunoprecipitation analysis, we show that MSP58 binds to importin α1 and α6, suggesting that nuclear targeting of MSP58 utilizes a receptor-mediated and energy-dependent import mechanism. Functionally, our data show that both nuclear and nucleolar localization of MSP58 are crucial for transcriptional regulation on p21 and ribosomal RNA genes, and context-dependent effects on cell proliferation.

**Conclusions:**

Results suggest that MSP58 subnuclear localization is regulated by two nuclear import signals, and that proper subcellular localization of MSP58 is critical for its role in transcriptional regulation. Our study reveals a molecular mechanism that controls nuclear and nucleolar localization of MSP58, a finding that might help future researchers understand the MSP58 biological signaling pathway.

**Electronic supplementary material:**

The online version of this article (doi:10.1186/s12929-015-0136-0) contains supplementary material, which is available to authorized users.

## Background

The active transport of macromolecules into the nucleus occurs through the nuclear pore complex (NPC) in the nuclear envelope. This process is mediated by specific signals called nuclear localization sequences (NLSs). ‘Monopartite’ NLSs contain a single stretch of 4–6 arginines or lysines; ‘bipartite” localization signals contain two stretches of basic amino acids separated by a spacer of 10–12 amino acids; and, most recently classified, ‘tripartite’ NLSs comprised of three clusters of two or three consecutive basic amino acid residues separated by two spacer peptides [1-7]. Proteins containing classical NLS sequences are imported into the nucleus by a heterodimeric receptor complex composed of importin α and β. Importin α proteins recognize and bind to the NLS, while importin β mediates the binding of the transport complex to the NPC. The delivery of cargoes and recycling of transport receptors occurs in an energy-dependent mechanism [8].

Eukaryotes have a specialized nuclear compartment, the nucleolus, which is a highly organized and dynamic non-membrane-bound subcompartment of the nucleus. The nucleolus is the site for ribosomal RNA (rRNA) synthesis and ribosome biogenesis; it is composed of multiple protein-protein and protein-nucleic acid interactions that are constantly changing in response to the differing metabolic conditions of the cell [9-11]. There are three major components of the nucleolus: the fibrillar center (FC), the dense fibrillar component (DFC), and the granular component (GC). The processing of rRNA is spatially arranged in accordance to the ultrastructure of these compartments [10,12]. The nucleolus, as accumulated studies suggest, also plays critical roles in regulating cell proliferation, senescence, stress sensing, signal recognition particle (SRP) assembly, the modulating telomerase function, and tumor suppression and oncogenic activities [13-15]. Some of these functions are mediated through the sequestration or release of transcription factors that control the cell cycle [15-17]. While active transport mechanisms are required for nuclear localization, nuclear proteins pass through the nucleolus randomly, and those with an affinity to constitutive nucleolar constituents are retained. Nucleolar localization signals (NoLSs) have been shown to represent binding domains with resident nucleolar proteins, rRNA, and other nucleolar components, all functioning more as retention rather than targeting or transport signals [16,18-23]. However, there is no consensus on NoLS sequence or structure [24-27]. Many NoLSs are usually rich in arginine and lysine residues, which overlap with NLSs (reviewed in [12]).

The 58-kDa microspherule protein (MSP58, also known as MCRS1) is one of these important nucleolar components that contributes to a number of cellular processes, including regulation of transcription through its interactions with various transcription factors [28-31]. Initially identified as an interaction partner of the proliferation-related nucleolar protein p120 [32], MSP58 was further shown to behave as an oncogene in fibroblast transformation assays, as does its quail homologue TOJ3 [33,34]. Interestingly, we found that MSP58 directly interacts with BRG1 and modulates the p53/p21 senescence pathway [35]. Our research also shows that MSP58 represses telomerase activity by inhibiting TEIF-mediated transactivation of the *hTERT* promoter [31]. In addition, as we previously reported, MSP58 can interact with, and relieve, the transcriptional repressor activity of Daxx through a nucleolar sequestration mechanism [29]. MSP58 also recruits the protein FMRP Iso6 to the nucleolus, an interaction that may contribute to neuronal translation regulation [36]. MSP58 also interacts with UBF, Mi-2β, and RET Finger Protein (RFP) in the nucleolus, and up-regulates ribosomal gene transcription [30]. One putative NoLS (amino acids 44–56) and a NLS (amino acids 113–123) of MSP58 have been previously predicted [32]; however, their functionality has not been experimentally confirmed.

In this study, we investigated the regulatory signals that determine nuclear and/or nucleolar localization of MSP58. Our results clearly defined two separate NLSs as responsible for MSP58 nuclear localization, and the N-terminal one also acts as a NoLS. Furthermore, identification of importin α1 and α6 as MSP58 partners was demonstrated. Finally, we provide evidence to support an essential role of nuclear and nucleolar localization for the biological function of MSP58.

## Methods

### Plasmids and antibodies

In our previous studies, we employed yeast constructs expressing LexA-MSP58 and its deletion mutants, LexA-MSP58 1–300 and LexA-MSP58 300–462, along with the mammalian vector expressing EGFP-MSP58 [31]. In order to generate MSP58 deletion mutants for expressing LexA fusion in yeast, we inserted polymerase chain reaction (PCR)-generated cDNA fragments encoding MSP58 amino acids 1–100 and 102–300 into the pBTM116 vector. To generate mammalian expression constructs of EGFP-fused MSP58 deletion mutants, we inserted PCR-generated cDNA fragments encoding MSP58 amino acids 1–300, 1–100, 102–300 and 300–462, into the pEGFP-C2 vector (BD Biosciences Clontech). We generated HA-MSP58 by cloning the full-length MSP58 (amino acids 1–462) into the pcDNA3.1-HA expression vector (Invitrogen). Using a Quikchange site-directed mutagenesis kit (Stratagene) employing pBTM-MSP58, pcDNA3.1-MSP58 and pEGFP-MSP58 as templates, we created the MSP58 mutation at lysine or arginine residue, or a series of the MSP58 NLSs mutants in the pBTM116, pcDNA3.1-HA and pEGFP-C2 vectors. Plasmids pACT2-importin α6; pACT2-importin α1 and pACT2-importin α3 were kind gifts from Dr Jero’ nimo Bravo (Centro Nacional de Investigaciones Oncolo’ gicas, Madrid, Spain). To generate Gal4 AD-importin β, we cloned a full-length importin β into the pACT2 vector (BD Biosciences Clontech). The luciferase reporter plasmid, prHu3-Luc, as previously described [37], was a gift from Dr. Yan-Hwa Wu Lee (National Yang-Ming University, Taipei, Taiwan). The original bacterial expression construct encoding GST-importin α1 [38] was a kind gift from Dr. Yoshihiro Yoneda (Osaka University, Japan). For the GST-fusion construct of importin α6, wild-type importin α6 was amplified by PCR and cloned into the EcoRI and XhoI sites of pGEX-4T2 to generate full-length GST-importin α6. The pSUPER-MSP58 construct and rabbit MSP58 antibody were described previously [35]. We verified all plasmids by restriction enzyme digestion and DNA sequencing analyses. In this study we used the following commercial antibodies: HA (HA.11; Babco/Covance), Importin α1 (ab84440; Abcam), Importin α3 (GTX 106325; GeneTex), Importin α6 (GTX 112203; GeneTex), Importin β (GTX 22811; GeneTex), GFP (JL-8, Clontech), p53 (BP53-12; Upstate Biotechnology), p21 (05–345, Upstate Biotechnology), and actin (clone AC-74; Sigma).

### NLS prediction

We employed a program, PSORTII (psort.nibb.ac.jp), designed to predict the protein sorting signals and the localization sites, to predict the potential NLSs of MSP58.

### Yeast two-hybrid screen and β-galactosidase assay

We used the LexA-MSP58 construct to screen the human testis cDNA library (Clontech). The yeast two-hybrid screening and analysis has been described previously [35]. In brief, we first transformed the L40 yeast strain with the LexA-MSP58 plasmid followed by transformation with 100 μg of the cDNA library. Yeast transformants were selected for protein interactions on medium lacking histidine, leucine, and tryptophan. Histidine protrotophic (His^+^) colonies were further tested for β-galactosidase activity. The plasmids from both His^+^ and X-gal^+^ colonies were isolated and sequenced. We performed quantitative liquid β-galactosidase assays using lysates from three separate yeast cultures according to the instructions of the Galacto-light Plus kit (Tropix).

### Cell lines, transfection and growth curves

COS-1 and HeLa cells were cultured in Dulbecco’s modified Eagle’s medium (DMEM; Invitrogen) supplemented with 10% fetal bovine serum (FBS; Invitrogen). HT1080 cells were maintained in a minimum essential medium (MEM; Invitrogen) with 10% FBS; all cells were kept at 37°C in a 5% CO_2_ atmosphere. Transfection of plasmids was by a PolyJet™ reagent (SignaGen Laboratories). In order to establish stable high expression cell lines, we selected cells with 200 μg/ml neomycin (G418). Neomycin-resistant cells were pooled for subsequent analysis. We monitored the growth rate of HT1080 and HeLa cells stably expressing HA-tagged wild type MSP58 or NLS mutants of MSP58 by seeding 2X10^5^ cells in 60-mm dishes containing 5% FBS, taking daily samples and counting them with a hemocytometer.

### Western blot, coimmunoprecipitation, and luciferase analyses

The transfected COS-1, HT1080 and HeLa cells were lysed in a modified RIPA buffer that consisted of 50 mM Tris–HCl, pH 7.8, 150 mM NaCl, 5 mM EDTA, 0.5% Triton X-100, 0.5% Nonidet-P40, 0.1% sodium deoxycholate, and a protease inhibitor mixture (Complete, Roche Molecular Biochemicals). The lysates were then subject to immunoprecipitation and Western analyses. To test the interaction in mammalian cells, cell lysates from stable HT1080 cells expressing HA-tagged MSP58 were mixed with anti-serum against HA, and anti-HA immunocomplexes were then collected by protein A/G PLUS-agarose beads (Millipore). The immunocomplexes were then subject to Western blot analysis with the anti-HA antibody or with the anti-importin α1 antibody. For the reporter assays, cells were cotransfected with prHu3-Luc and the internal control reporter pRL-TK. Cells were harvested at 48 hours post-transfection, with firefly luciferase activity measured by the dual-luciferase reporter assay system (Promega) and normalized against Renilla luciferase activity.

### Immunohistochemistry and immunofluorescence

Tissue samples from patients with aggressive human oral cavity squamous cell carcinoma (OCSCC) were examined by immunohistochemical staining. Clinicopathological information for each patient, including sex, age, tumor stage, nodal status and TNM stage, was obtained retrospectively from clinical records and pathological reports. This study was approved by the Medical Ethics and Human Clinical Trial Committee at Chang Gung Memorial Hospital, Taiwan. Tissues were fixed with 10% buffered formalin embedded in paraffin and decalcified in 10% EDTA solution. Representative blocks of the formalin-fixed, paraffin-embedded tissues were cut to 4 mm and deparaffinized with xylene and rehydrated in a series of ethanol washes (100, 90, 80, and 70%). Slides were washed with phosphate-buffered saline (PBS) and treated with 3% H_2_O_2_ for 30 minutes to block endogenous peroxidase activity. Next, the sections were microwaved in 10 mM citrate buffer, pH 6.0, to unmask the epitopes. Immunohistochemical staining was performed as previously described [39]. After antigen retrieval, we incubated the sections with diluted anti-MSP58 polyclonal antibody at room temperature for 1 hour, followed by washing with PBS. We then applied horseradish peroxidase/Fab polymer conjugate (PicTure™-Plus kit; Zymed) to the sections for 30 minutes, and then washed them with PBS. Finally, we incubated the sections with diaminobenzidine for five minutes to develop the signals. Indirect immunofluorescence staining on the HeLa cell lines was performed with anti-MSP58 polyclonal antibody at room temperature for one hour. The slides were then washed with PBS and incubated with FITC-conjugated goat anti-rabbit IgG (Jackson ImmunoResearch) at room temperature for one hour. DAPI staining was applied to stain the nuclei. After washing again with PBS, we mounted the sections with GEL/Mount (biomeda corp) to prepare the fluorescence images for analysis with an Olympus BX51 microscope.

### GST pull-down assays

The MSP58 was expressed in vitro using plasmid containing a T7 promoter in a rabbit reticulocyte lysate system (Promega). The recombinant GST, GST-importin α1 and α6 proteins were purified from lysates of *Escherichia coli* BL21 cells with glutathione-Sepharose 4B (GE Healthcare) under native conditions following the protocols described before [40]. The reaction mixtures (40 μl) containing MSP58 proteins from a cell-free transcription and translation system were incubated for one to two hours with 2 μg of GST and GST-importin α1 or α6 fusion proteins in 0.2 ml of a binding buffer (10 mM Hepes (pH 7.5), 50 mM NaCl, 0.1% Nonidet P-40, 0.5 mM dithiothreitol, and 0.5 mM EDTA), followed by pull-down with glutathione Sepharose, wash three times in binding buffer, and analysis by sodium dodecylsulfate (SDS)-polyacrylamide gel electrophoresis (PAGE) and Western blotting using an anti-HA antibody. GST and GST fusion proteins in the reaction mixture were visualized by Coomassie blue staining.

### Analysis of senescence

We performed SA-β-gal staining using a Senescence Detection kit (Cell Signaling Technology).

### Isolation of RNA and real-time quantitative PCR (qPCR)

RNA isolation and the reverse-transcription (RT)-PCR were performed as previously described [31,35]. The real-time qPCR was performed using the SYBR Green Advantage qPCR Premix (Clontech) and C1000™ Thermal Cycler (Bio-Rad Laboratories). PCRs were performed using the following conditions for 40 cycles: 95°C for 15 seconds, 60°C for 15 seconds, and 72°C for 20 seconds.

We used the following primer pairs: p21, forward (5′-ATGTGGACCTGTCACTGTCTTG) and reverse (5′-CGTTTGGAGTGGTAGAAATCTG); 47S ribosomal gene, forward (5′-CCT GTC GTC GGA GAG GTT GG) and reverse (5′-ACC CCA CGC CTT CCC ACA C); and GAPDH, forward (5′-CCCACTCCTCCACCTTTGAC-3′) and reverse (5′-TCTCTCTTCCTCTTGTGCTCTTG-3′).

### Chromatin immunoprecipitation (ChIP) analysis

ChIP assays were performed with a ChIP assay kit (Millipore) according to the manufacturer’s protocol. Briefly, sheared chromatin fragments were immunoprecipitated with anti-HA antibody, or control immunoglobulin G (IgG) at 4°C overnight. After dissociating the DNAs from the immunoprecipitated chromatin, we PCR-amplified the DNAs. The association of HA-MSP58 proteins with the 47S ribosomal promoter region was measured with a PCR using the following primers: forward 5′-CCCTGCGTGTGGCACGGGC and reverse 5′-AGGAGCGCGGCCGGCTAGCC. The amplified DNA was separated on 2% agarose gels and visualized by ethidium bromide staining.

### Statistical analysis

We performed statistical comparisons using a two-tailed Student’s *t* test. The P-values were calculated using the GraphPad Prism software version 3.03 package (GraphPad Software, San Diego, CA).

## Results

### Nucleolar localization of MSP58

**Figure 1:**
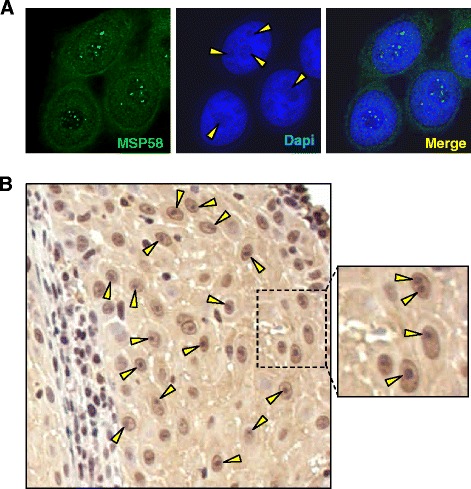
Subcellular distribution of MSP58. **A**. Immunocytochemical staining of HeLa cells using a MSP58 polyclonal antibody. MSP58 was visualized using a secondary antibody conjugated to FITC (green), and nuclei were counterstained with DAPI (blue). Nucleoli are visible as dark regions in the nuclei (arrowhead). Note the presence of several microspherules in the nucleoli. **B**. Immunohistochemical staining of MSP58 protein expression in a tissue of human oral cavity squamous cell carcinoma (OCSCC). Images were analyzed using an Olympus BX51 microscope with 200× magnification. Arrowheads indicate nucleoli.

Our previous studies have shown that the endogenous MSP58 protein is localized in the nuclei and the microspherule speckles of nucleoli in HeLa cells ([35] and Figure 1A). In addition, we also detected MSP58 expression profiles using a tissue microarray by an immunohistochemical (IHC) analysis [35]. Interestingly, we found strong MSP58 nuclear and nucleoli staining in some tumor tissues including a tissue specimen of human oral cavity squamous cell carcinoma (OCSCC) (Figure 1B). MSP58 is thus considered to belong to a nucleolar protein family member as described previously [32]. Since the mechanism of MSP58’s translocation into the nucleus/nucleolus is not entirely known, we began with the identification of its NLS motif(s).

### Determination of nuclear localization sequences in MSP58

**Figure 2:**
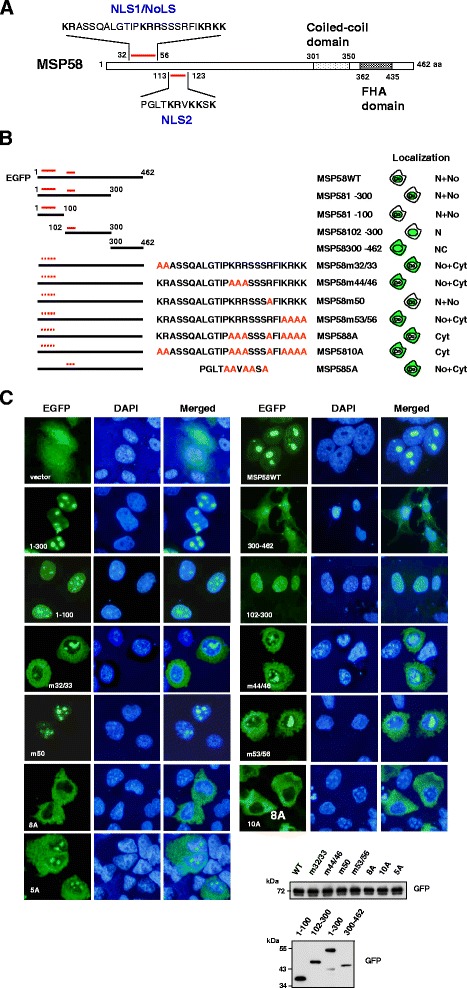
Characterization of two nuclear localization signals in MSP58. **A**. Schematic diagrams of putative nuclear localization sequences (NLS1 and NLS2) in MSP58. Charged, basic amino acid residues are indicated in boldface. **B**. Schematic representation of the different EGFP-MSP58 fusion proteins tested and their localization pattern within cells. The point mutations were introduced at the two NLS loci by replacing the basic amino acid residues with alanine residues. The patterns were represented as being predominantly nuclear (N), nuclear and nucleolar (N + No), even throughout the entire cell (NC), cytoplasmic (Cyt), or nucleolar and cytoplasmic (No + Cyt). **C**. COS-1 cells were transfected with EGFP and EGFP-MSP58 mutant constructs as indicated in the figure and fixed 24 hours after transfection. The green color showed the fluorescence of the EGFP-fusion protein. Corresponding DAPI images are also shown. Total cell lysates from COS-1 transfected cells with the indicated constructs were analyzed by Western blotting using an anti-GFP antibody.

Previous reports have predicted one putative NoLS (amino acid residues 44–56) and one NLS (residues 113–123) of MSP58 [32]; however, prior studies were unable to confirm the function of these sequences [32,33,36]. Our use of a PSORT II computer program to analyze MSP58 sequences identified three putative NLSs, two monopartite signals (PKRR; aa 43–46, and KRKK; aa 53–56) and a bipartite signal (PGLTKRVKKSK; aa 113–123). The PSORT program did not indicate any nuclear export signals (NES) within MSP58 (data not shown). We noted that the motif encompassing of residues 32–56 in MSP58 is a dual bipartite motif, which contains one bipartite motif KRASSQALGTIPKRR, starting at residue 32 and ending at residue 46, and another bipartite motif KRRSSSRFIKRKK starting at residue 44 and ending at residue 56, whereas the motif encompassing residues 113–123 contains a monopartite signal (PGLTKRVKKSK) (Figure 2A). Moreover, both the NLS motifs were highly conserved in the MSP58 family of proteins, p78 and MCRS2 (Additional file 1). This pattern held across diverse species such as human, mouse, xenopus, zebrafish and quail, which highlights the potential of NLSs to regulate MSP58 nuclear translocation. Therefore, we designated the regions from residues 32 to 56 as NLS1, and 113 to 123 as NLS2.

To study the function of these putative NLSs, we evaluated the localization patterns of various MSP58 deletions or MSP58 NLS mutants fused to EGFP after transient expression in COS-1 cells (Figure 2B and C). The expression levels of the different mutants were confirmed by Western blotting (Figure 2C). The EGFP control vector showed an overall homogenous distribution pattern. Like full-length MSP58, the N-terminal fragment of MSP58 construct (amino acids 1–300) allows the efficient import of chimeric proteins into the nuclei and nucleoli (Figure 2C). Thus it seems reasonable to conclude that the N-terminal dual bipartite and monopartite motifs may serve as NLSs. In contrast, the C-terminal fragment of MSP58 construct (amino acids 300–462) was not accumulated in the nucleus or in nucleoli, and it exhibited a localization rather comparable to that of the EGFP alone. Also, EGFP-MSP58 1–100 fusion protein containing NLS1 motif localized efficiently to the nucleus and clearly accumulated in the nucleoli. In contrast, the fragment 102–300 of MSP58 containing NLS2 motif, can drive nuclear localization of EGFP but cannot drive nucleolar accumulation (Figure 2C). Furthermore, we mutated the two NLS motifs by changing the basic residues in the individual NLS or all of the basic residues in the two NLSs shown in Figure 2B to alanine residues. Remarkably, mutations in any one of the three clusters of the NLS1 or monopartite NLS2 (m32/33, m44/46, m53/56 and 5A) showed an increased cytoplasmic expression in addition to expression in the nucleoli (Figure 2B). The results indicated that both of these stretches of basic residues are critical for nuclear import. Mutation of the arginine amino acid at residue 50 in NLS1(m50) had no effect on MSP58 nuclear/nucleolar localization. Notably, combinatorial mutations of both the second and third basic amino acid clusters of the tripartite NLS1 (8A) completely eliminated both nuclear and nucleolar accumulation (Figure 2C), suggesting that residual 44–56 within NLS1 also functions as a (bipartite) NoLS. Furthermore, the three clusters of basic residues triple mutant (10A) display a similar phenotype as the 8A mutant (Figure 2C), a result were also shown in a number of different cells, including HeLa and MCF7 (data not shown). Together these results suggest that both NLS1 and NLS2 can contribute to the nuclear import of MSP58 and that NLS1 also functioned as a NoLS.

### MSP58 interacts preferentially with importin α1 and importin α6

**Figure 3:**
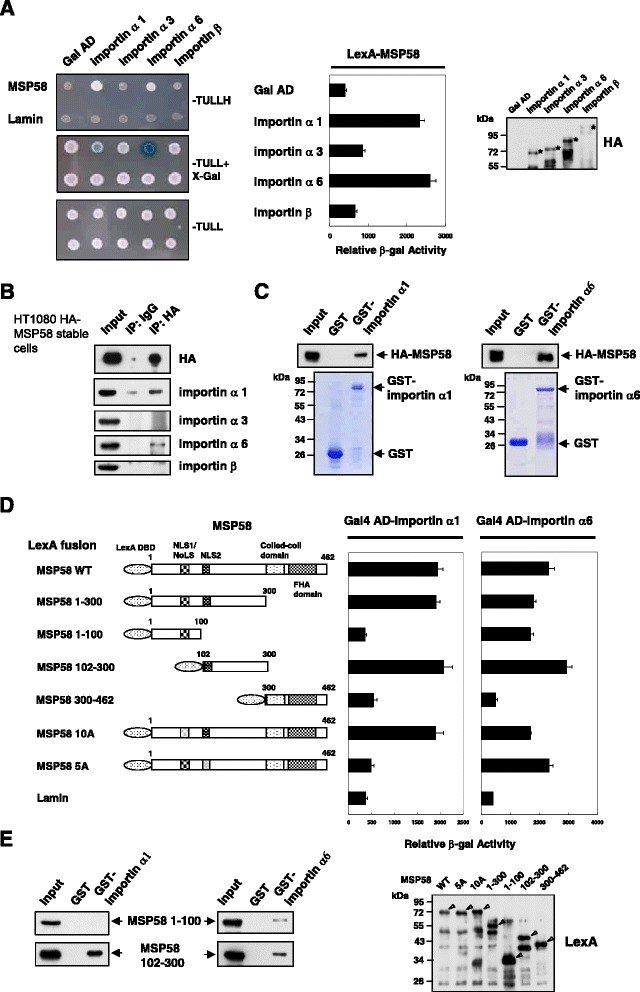
MSP58 interacts with importin α1 and α6. **A**. Interaction of MSP58 with importins in a yeast two-hybrid assay. Yeast transformants with bait and prey as indicated were spotted on histidine-containing (−TULL), without histidine (−TULLH), and both histidine- and X-Gal-containing (−TULL + X-Gal) media (left) in plates. LexA-lamin served as a negative control. Yeast cotransformed were analyzed by quantitative β-Gal assays (middle). Data represent the mean ± the standard deviation of three separate experiments. A Western blot shows expression levels of different importins in yeast cells (right). **B**. Coimmunoprecipitation assays. Whole cell lysates from HT1080 cell lines stably expressing HA-MSP58 were subjected to immunoprecipitation (IP) experiments followed by a Western blot analysis with the indicated antibodies. IP by mouse immunoglobulin G (IgG) was used as a negative control. **C**. GST and fusion proteins GST-importins α1 and α6 were expressed in *Escherichia coli* BL21 cells and immobilized on glutathione-Sepharose. The MSP58 was obtained with a cell-free transcription and translation system *in vitro* and incubated with GST, GST-importin α1, or α6 proteins. Bound proteins were detected by immunoblotting with an anti-HA antibody. Coomassie blue-stained gel shows the input of GST-fusion proteins used. **D**. Schematic presentation of wild-type and different mutants of MSP58 tested in yeast two-hybrid assays (left). Numbers indicate the amino acid position. Yeast cotransformed with bait and prey as indicated were analyzed by quantitative β-Gal assays (right). Data represent the mean ± the standard deviation of three separate experiments. LexA-lamin served as a negative control. Immunoblotting shows the expression levels of different LexA-MSP58 fusion proteins in yeast. **E**. GST and GST-importin α1 or α6 proteins were immobilized on glutathione-Sepharose and incubated with cell lysates containing EGFP MSP58 1–100 or EGFP MSP58 102–300. Bound proteins were resolved by SDS–PAGE followed by Western blot analysis using anti-GFP antibodies.

To identify proteins that regulate MSP58 nuclear expression, we performed yeast two-hybrid screening using the full-length MSP58 as a bait. One positive clone encoding importin α1/karyopherin α2/Rch1 cDNA, amino acid 150–592, was isolated from a Matchmaker human testis cDNA library (data not shown). Different importins, also named karyopherins (KPNAs), are known to bind NLS-bearing cargoes in the cytoplasm and transport them into the nucleus. To characterize the transport receptor for MSP58, we first analyzed by a yeast two-hybrid assay the interaction of MSP58 protein with several importins. The S. cerevisiae L40 strain was cotransformed with a yeast vector containing the MSP58 full-length sequence (BTM-MSP58) and one of the yeast expression plasmids encoding the entire importin α1 (pACT2/KPNA2), importin α3 (pACT2/KPNA4), importin α6 (pACT2/KPNA5) or importin β. After 4–5 days of incubation, co-transformed cells overexpressing importin α1 (KPNA2) and importin α6 (KPNA5) were capable to grow (Figure 3A). However, yeast colonies overexpressing importin α3 (KPNA4) or importin β failed. The interaction was further verified by colony and liquid β-galactosidase assays. The interaction between MSP58 and importin α1 or α6 was further validated by co-immunoprecipitation assays in mammalian cells. Endogenous importin α1 and α6 proteins were coimmunoprecipitated in the HT1080 stable cell lines expressing HA-tagged MSP58 (see below) by an anti-HA antibody (Figure 3B). To further verify the interaction between MSP58 and importin α1 or α6 in vitro, we performed GST pull-down experiments using the GST-importin α1 or α6 fusion protein and in vitro-translated MSP58 protein. As shown in Figure 3C, the full-length MSP58 was specifically pulled down by GST-importin α1 or α6, but not by the GST protein.

To further confirm the functional NLSs of MSP58, we tested the specificity of the interaction of MSP58 and importin α1 or α6 using the yeast two-hybrid β-galactosidase liquid assay. To do this, we fused either full length, deleted, or NLSs point-mutated clones of MSP58 to the LexA DNA-binding domain in BTM116 (Figure 3D). In addition, we fused a full-length of importin α1 or α6 to the activation domain of GAL4 in the pACT2 vector. In this assay, importin α1 interacted with full-length MSP58, MSP58 1–300 and MSP58 102–300, but not with MSP58 1–100 and MSP58 300–462 (Figure 3D), a pattern that indicates the importin α1 binding site is located at aa 102–300 of MSP58. Consistently, the NLS1 mutant (10A) of MSP58 maintained the ability to interact with importin α1, whereas the NLS2 (5A) mutation disrupted the interaction of MSP58 with importin α1. These results demonstrate that MSP58 binds to importin α1 through the monopartite NLS2. In contrast, importin α6 interacted strongly with full-length MSP58, MSP58 1–300, 1–100, 102–300 fragments and NLS mutants (both 5A and 10A), but not with MSP58 300–462 fragment, suggesting that importin α6 binds to both NLS motifs (Figure 3D). These interactions were further confirmed by GST pull-down assays. COS-1 cell lysates containing fusion proteins, EGFP-MSP58 1–100 and EGFP-MSP58 102–300 were mixed with glutathione-Sepharose beads that had been pre-bound to either GST or GST-fused importin α1 or α6 proteins. The bound proteins were examined by SDS-PAGE followed by Western blotting using anti-GFP antibodies. As shown in Figure 3E, GST did not bind importin α1 or α6, while MSP58 1–100 interacted specifically with importin α6, but not with importin α1. MSP58 102–300 on the other hand, bound both importin subtypes. These results consistently demonstrate that MSP58 binds selectively to importin α1 and importin α6 through both NLS1 and NLS2.

### Effect of MSP58 NLSs on the control of gene expression and cell proliferation

**Figure 4:**
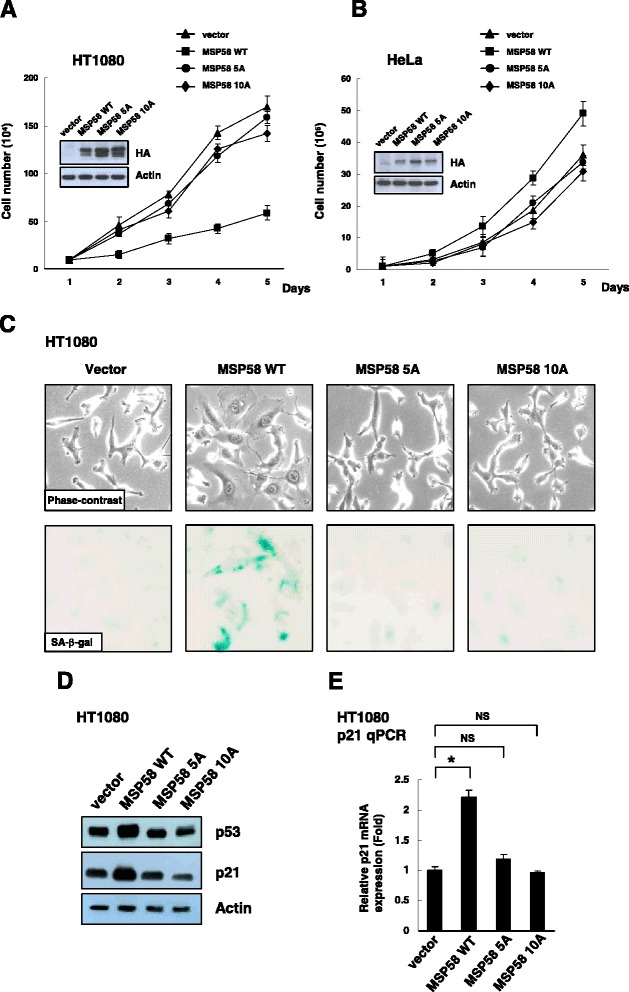
Effects of mutation of MSP58 NLSs on p21 gene expression and cell proliferation. **A** and **B**. Growth curves of HT1080 and HeLa, stable control (vector); wild type; NLS1 mutation (10A) and NLS2 mutation (5A) MSP58-overexpressing cells. Points, mean value from three independent experiments, each run in triplicate; bars, S.D. MSP58 expression was analyzed using immunoblotting. **C**. Phase-contrast images and SA-β-gal activity of control vector and MSP58 overexpression HT1080 stable cell lines. Images are all at the same magnification (200×). **D**. Total cell extracts were analyzed using immunoblotting with the indicated antibodies. **E**. p21 mRNA expression was analyzed by quantitative RT-PCR using GAPDH levels as the internal control. The expression of p21 in HT1080 control vector cells was defined as 1.0, and other values were normalized accordingly. Columns, mean of three independent experiments; bars, S.D. *, p < 0.05. NS, non-significant.

We have previously shown that the overexpression of MSP58 induces context-dependent roles in controlling cell proliferation [35].In particular, MSP58 induces cellular senescence through p53/p21-dependent pathway. Since the 10A/NLS1 and 5A/NLS2 mutations disrupt the nuclear localization of MSP58 (Figure 2), they provide a tool to test whether nuclear localization is required in MSP58-regulated gene expression and cell proliferation. We further established HT1080 and HeLa cell lines with stable expression of ectopic HA-tagged wild-type or NLS mutant (5A or 10A) MSP58. Protein expression was confirmed by immunoblotting using the anti-HA antibody. HT1080-derived cell lines that stably expressed wild-type MSP58 consistently revealed senescence-like phenotypes typified by enlarged and flattened cells, SA-β-Gal positive, and a slower growth rate than that of vector control cells (Figure 4*A* and *C*). In contrast, HeLa cells with stable MSP58 overexpression proliferated more rapidly and exhibited no morphological differences compared with control cells (Figure 4*B* and data not shown). Interestingly, this cell-proliferative effect was eliminated in the both 10A/NLS1 and 5A/NLS2 mutants, suggesting that nuclear and/or nucleolar localization is important for the cell growth control of MSP58. As expected, we found increased expression of p53 and p21 in HT1080 cells transfected with wild-type MSP58, whereas no obvious effect was shown in HT1080 cells transfected with NLSs mutants MSP58 (Figure 4D and E). These results suggest that the NLS motifs and nuclear/nucleolar localization are important for MSP58 in controlling gene expression and cell proliferation.

### Importance of the MSP58 NoLS for ribosomal gene transcription

**Figure 5:**
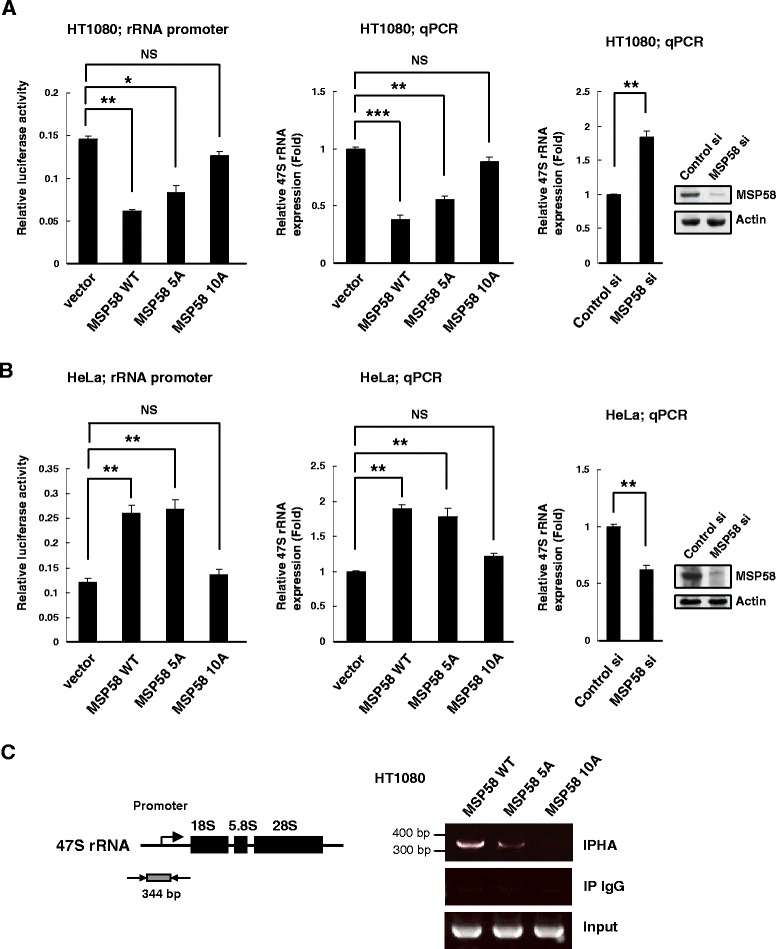
Effects of mutation of MSP58 NLSs on ribosomal gene transcription. **A** and **B**, pol I-dependent transcription of prHu3-Luc reporter (left). prHu3-Luc reporter plasmid (firefly) and the internal control plasmid pRL-TK (Renilla) were co-transfected into HT1080 or HeLa stable cell lines as indicated. After 36 h, both firefly and Renilla luciferase activities were measured. Columns, mean of three independent experiments; bars, S.D. *, p < 0.05. **, p < 0.01. NS, non-significant. Quantitative RT-PCR analysis of 47S rRNA HT1080 or HeLa stable cell lines (middle). Values for 47S rRNA were normalized to the GAPDH housekeeping control. The expression of 47S rRNA in vector control cells was defined as 1.0, with other values were accordingly normalized. Columns, mean of three independent experiments; bars, S.D. **, p < 0.01. ***, p < 0.001. NS, non-significant. (Right) HT1080 and HeLa cells were transfected with control or MSP58-knockdown (pSuper-MSP58 si-3) plasmid. At 48 h after transfection the expressions of 47S rRNA were analyzed by quantitative RT-PCR. GAPDH was amplified as a control. MSP58 protein levels were assessed by Western blotting. Columns, mean of three independent experiments; bars, S.D. **, p < 0.01. **C**. Schematic representation of the 47S gene organization and position of the specific primers used for ChIP assays (left). HT1080 cells stably expressing wild-type and NLS mutants (5A and 10A) MSP58 were fixed with formaldehyde, and cross-linked chromatin was precleared and immunoprecipitated with anti-HA antibody or normal IgG (negative control) as indicated. Eluted DNA was analyzed by a PCR using primers corresponding to the 47S gene promoter sequence, and the resultant DNA fragments of 344 bp were separated on 2% agarose gels. “Input” bands were obtained from DNA purified from chromatin not yet immunoprecipitated. Experiments were repeated three times.

It has been shown that MSP58 co-localizes with rRNA transcription factor UBF in nucleoli and positively regulates ribosomal RNA (rRNA) gene transcription [30]. We further compared the wild-type and NLS mutants of MSP58 on rDNA promoter activity and rRNA expression. The human rRNA promoter luciferase reporter plasmid prHu3-Luc has been applied to measure Pol I transcription activity [37]. Transcription of the prHu3-Luc reporter was inhibited in HT1080 cells stably expressing wild-type MSP58 as compared with control cells, a pattern that coincided with a reduction of 47S mRNA levels (Figure 5A). Contrariwise, both rDNA promoter activity and 47S rRNA expression were activated in MSP58 stable HeLa cells (Figure 5B). The MSP58 5A/NLS2 mutant showed slightly reduced effects on rRNA transcription in both HT1080 cells and HeLa cells as compare to the wild-type MSP58. On the contrary, the 10A/NLS1 mutant failed to modulate rRNA gene transcription in either HT1080 or HeLa cells, suggesting that nucleolar localization of MSP58 is important for the regulating rDNA transcription (Figure 5A and B). Additionally, knockdown of MSP58 expression in HT1080 cells resulted in increased 47S mRNA expression (Figure 5A). In contrast, knockdown of MSP58 expression decreases 47S mRNA levels in HeLa cells (Figure 5B). These results indicate that MSP58 may act in a context-dependent manner to regulate rDNA transcription, a finding consistent with our previous studies [31,35]. Furthermore, ChIP assays were performed on the rDNA promoter to establish the occupancy of wild-type and NLS mutants MSP58. Chromatin was immunoprecipitated from MSP58 wild-type, 5A, and 10A expressing HT1080 cells with HA and non-specific IgG antibodies, and the rDNA was amplified with primers specific for the promoter [30]. This analysis revealed that wild-type MSP58 binds to the rDNA promoter, whereas the 5A/NLS2 mutant showed reduced binding to the rDNA promoter (Figure 5C). However, the 10A/NLS1 mutant failed to interact with the rDNA promoter. These results suggest that the NLS1/NoLS motif or nucleolar localization is required for MSP58 to play its role in regulating the rDNA gene promoter activity.

## Discussion

The previous study reporting on MSP58 localization predicted that MSP58 contains a putative NoLS located between amino acids 44 and 56, and a classic NLS located between amino acids 113 and 123 [32]. We have established motifs in residues 32–56 (NLS1) and 113–123 (NLS2) as functional NLSs that decide nuclear localization of MSP58, a finding consistent with the prediction of the NLS motif (namely NLS2; residues 113–123). Notably, NLS1 has a nonclassical dual bipartite motif, whereas the NLS2 region contains a monopartite motif. In addition, one bipartite motif (residues 44–56; predicted NoLS) within the NLS1 appears to be essential for MSP58 nucleolar localization (Figure 2A). Other researchers have reported that MCRS2, encoding for an isoform of MSP58/MCRS1, contains a NLS sequence located at amino acids 66–69 (KRKK), which corresponds to amino acids 53–56 of MSP58, and they also found an NoLS located at amino acids 133 to 136 (KKSK), which corresponds to amino acids 120–123 of MSP58 [41]. The behavior of MSP58 m53/56 mutant supports this report by impairing its ability to enter the nucleus. However, our results differ in that we demonstrate the 5A mutant (comprising the KKSK motif) of MSP58 affected the nuclear translocation ability but has no effect on its nucleolar localization (Figure 2B and C). It is possible that the mechanisms of nuclear/nucleolar translocation of different MSP58 family members are diverse and may vary in different conditions or cell types. For NLS1, mutations in any one of these three clusters (m32/33, m44/46 or m53/56 mutant) affected the nuclear localization but had no effect on nucleolar localization activity of MSP58 (Figure 2). In addition, combinatorial mutations of second and third clusters of basic residues (amino acids 44–56; comprising the KRR and KRKK motifs) within NLS1 (8A mutant) resulted in a significant impairment of the nucleolar localization of MSP58. Consistently, mutations of all three clustered basic residues (10A mutant) led to an exclusively cytoplasmic localization of MSP58. These findings suggest that the minimal region required for nucleolar localization directed by NLS1 resides within residues 44–56. Most nuclear proteins are targeted to the nucleus by mono- or bipartite basic amino acid sequences in an NLS [42-44]. Although specific consensus sequences for NLS are not conserved, they generally tend to be short sequences rich in basic residues. Monopartite NLS sequences are characterized by a short single stretch of 4–6 basic residues, typified by the SV40 large T antigen NLS (PKKKRKV), whereas bipartite NLS sequences comprise two clusters of basic residues, separated by a spacer region of 10–12 residues (e.g., nucleoplasmin (KRPAATKKAGQAKKKK; [45]). In addition to these two types of NLSs, multiple non-classical NLSs have also been identified (reviewed in [46]); an example is the tripartite NLS in EGFR family members [4]. The novel NLS1 of MSP58 we identified contains three clusters of basic amino acids. In addition, both the NLSs were highly conserved among MSP58 proteins from different species and among different MSP58 family proteins (p78 and MCRS2) (Additional file 1), suggesting that the same motifs may be responsible for nuclear translocation of the other MSP58 family proteins. Our findings reveal the key determinants of nuclear and nucleolar localization of MSP58.

The mechanism of protein localization to the nucleolus is still not clearly understood. Perhaps the nucleolar targeting of proteins is related to direct or indirect interaction with certain nucleolar components such as rDNA, rRNA, or nucleolar proteins [11,47]. Thus, it is uncertain whether nucleolar proteins are specifically localized, targeted, accumulated or just retained in this compartment. Even though numerous studies of cellular and viral proteins harbouring NoLS(s) have been reported in recent years, and it is known that NoLSs are usually rich in positively-charged amino acids such as lysine and arginine residues, there is still no obvious consensus on sequence or structure [24-27]. Proteins that localize in the nucleoli can also have nuclear import and export motifs. It has been suggested that proteins localize in the nucleoli must first be imported into the nucleus and therefore they likely possess both NLS and NoLS whose sequences may overlap (reviewed in [12]), and our results support this notion. Our data suggest that NLS1 of MSP58 also functions as a NoLS. Some examples show that the NoLSs are part of the NLSs. The 18-kDa FGF2 isoform contains two NLSs, with the carboxy-terminal also acting as a NoLS [48]. The H3N2 subtype influenza A virus NS1A protein contains two NLSs, the carboxy-terminal NLS2 in involved in nucleolar retention [49]. The putative tumor suppressor Parafibromin contains a bipartite NLS and three distinct NoLSs, all of which are critical for its nucleolar localization [50]. These examples highlight the direct link between the nucleolar targeting process and the nuclear import process.

A variety of proteins are now known to contain two or more nuclear localization signals. Our data suggest that the mutation of either NLS is sufficient to impair MSP58 nuclear translocation, resulting in the cytoplasmic localization of the protein and a loss of gene expression from the p21 promoter. The existence of multiple NLSs within a single protein may provide multiple mechanisms to exploit different targeting controls for proteins whose nuclear localization is critical for function [51]. It is likely that the existence of multiple NLSs can increase the likelihood of transporter interaction to ensuring a more efficient coordination of translocation. Six different isoforms of importin α have been described in mammalian cells thus far [52] and there is evidence that different isoforms show significantly different affinities for specific NLS-containing substrates (reviewed in [53]). Furthermore, the binding specificity can be affected by abundance of nuclear import receptors. We identify importin α1 and α6 as novel proteins that bind significantly to MSP58. Our results suggest that importin α1 interacts directly with the NLS2 sequences of MSP58, while importin α6 binds to both NLS1 and NLS2 (Figure 3). This leads us to hypothesize that MSP58 engages one or more transport receptors to enter the nucleus. Similarly, ribosomal proteins are preferentially transported into the nucleolus by more than one receptor [54]. We previously reported that replicative and stress-induced senescence increases MSP58 expression, and that MSP58 regulates senescence through p53/p21 pathway [35]. The regulation of MSP58 must be tightly controlled to effect cell cycle-related genes transcription at the proper time in response to cellular stimulation. We speculate that control of MSP58 translocation mediated by interaction with multiple importin family members may be one of a number of mechanisms by which cells carefully regulate MSP58 activity. Further studies are warranted to clarify whether other importin molecules participate in binding to MSP58 during its nuclear translocation.

Finally, we provide evidence that nuclear and nucleolar localization of MSP58 are essential for its transcriptional regulatory function and context-dependent effects on cell proliferation (Figures 4 and 5). The ribosomal gene expression and cell growth control activities of MSP58 were abrogated in cells expressing the nucleolar transport-defective MSP58. Chromatin immunoprecipitation (ChIP) assays revealed that MSP58 is recruited to the rRNA gene promoter and that an intact NLS1/NoLS is required for this binding and gene regulation (Figure 5C). MSP58 is reported to play a role in the activation of rRNA transcription [30]; our data, however, show that MSP58 expression regulates rRNA gene transcription in a cell context-dependent manner. The ectopic expression of MSP58 suppressed rRNA promoter activity and rRNA expression, whereas knockdown of MSP58 increased rRNA synthesis in HT1080 cells. In contrast, overexpression of MSP58 activated rRNA promoter activity and rRNA expression, while knockdown of MSP58 decreased rRNA synthesis in HeLa cells. As our previous studies showed that stable MSP58 overexpression promoted cell proliferation in HeLa cells, but induced cellular senescence in HT1080 cells [35], we found that MSP58 can play either a positive or a negative role in rRNA transcriptional regulation in different cell types. These findings reinforce the link between the level of transcription of rRNA genes and cell proliferation [55]. Nucleolar targeting of MSP58 suggests the possibility of its role in cell growth and proliferation by regulating ribosome biogenesis in the nucleolus. At present, however, the role of MSP58 in nucleolar chromatin dynamics remains unclear. MSP58 may participate in nucleolar processes such as transcription or it may recruit other cellular proteins and located there for temporal storage. This sequestration could prevent proteins from reaching their targets in chromatins or other cellular compartments. Our earlier investigation found that MSP58 interacts with Daxx and brings it to the nucleolus, thereby relieving its repression [29]. Notably, various oncogenes and tumor suppressors such as p53, p14 Arf, Rb, c-Myc, VHL, and PML have many different functions depending on their localization or sequestration [56-60]. These findings regarding nuclear import and nucleolar localization signals that control subcellular localization and transcriptional activity of MSP58 provide new insights toward a better understanding of MSP58 mediated oncogenic or tumor suppressive activity.

## Conclusions

In this study, we have identified and characterized two NLSs within MSP58: an unusual type of NLS (residues 32–56; NLS1) and a monopartite NLS (residues 113–123; NLS2). Notably, one bipartite motif (residues 44–56) within the NLS1 appears to be critical for MSP58 nucleolar localization. MSP58 selectively binds to importin α1 and α6 isoforms. Functionally, the MSP58 cytoplasmic mutant loses its ability to regulate gene expression and cell proliferation. Together, our findings provide new insights into the mechanism of the nuclear transport and nucleolar localization of MSP58.

## Additional file

Additional file 1:
**Sequence alignment of the MSP58 two NLSs across difference species, including human (h), mouse (m), Xenopus (x), zebrafish (z), quail (q) and Drosophila (d), and among members of the MSP58 family including p78 and MCRS2.**

## Abbreviations

MSP58: 58-kDa microspherule protein
NLS: Nuclear localization signal
NoLS: Nucleolar localization signal
rRNA: Ribosomal RNA
FC: Fibrillar center
DFC: Dense fibrillar component
GC: Granular component
TOJ3: Target of Jun 3
TEIF: Telomerase transcriptional element-interacting factor
SA-β-gal: Senescence-associated β-galactosidase
Daxx: Fas death domain-associated protein
X-Gal: 5-bromo-4-chloro-3-indolyl- β -D-galactopyranoside
qPCR: Quantitative PCR
DAPI: 4′,6-diamidino-2-phenylindole
