# Erratum: Identification and characterization of nuclear and nucleolar localization signals in 58-kDa microspherule protein (MSP58)

**DOI:** 10.1186/s12929-015-0153-z

**Published:** 2015-07-24

**Authors:** Chuan-Pin Yang, Chi-Wu Chiang, Chang-Han Chen, Yi-Chao Lee, Mei-Hsiang Wu, Yi-Huan Tsou, Yu-San Yang, Wen-Chang Chang, Ding-Yen Lin

**Affiliations:** Institute of Bioinformatics and Biosignal Transduction, College of Bioscience and Biotechnology, National Cheng Kung University, Tainan, 70101 Taiwan, ROC; Department of Pharmacology, College of Medicine, National Cheng Kung University, Tainan, 70101 Taiwan, ROC; Institute of Molecular Medicine, College of Medicine, National Cheng Kung University, Tainan, 70101 Taiwan, ROC; Infectious Diseases and Signaling Research Center, National Cheng Kung University, Tainan, 70101 Taiwan, ROC; Institute for Cancer Biology and Drug Discovery, College of Medical Science and Technology, Taipei Medical University, Taipei, 11031 Taiwan, ROC; Graduate Institute of Medical Sciences, College of Medicine, Taipei Medical University, Taipei, 11031 Taiwan, ROC; Center for Neurotrauma and Neuroregeneration, Taipei Medical University, Taipei, 11031 Taiwan, ROC; Program for Neural Regenerative Medicine, College of Medical Science and Technology, Taipei Medical University, Taipei, 11031 Taiwan, ROC; Center for Translational Research in Biomedical Sciences, Kaohsiung Chang Gung Memorial Hospital, Kaohsiung, 83301 Taiwan, ROC; Department of Applied Chemistry, National Chi Nan University, Puli, Nantou 54561 Taiwan, ROC

## Erratum

Due to a publisher error in the original version of this article [1], ‘8A’ was superimposed on the 10A – EGFP image in Fig. 2C. This error has now been corrected in the original.
