# Supplementary material for: Identification and characterization of nuclear and nucleolar localization signals in 58-kDa microspherule protein (MSP58)
Source: J Biomed Sci. 2015 May 16;22(1):33. doi: 10.1186/s12929-015-0136-0 (PMC4434885; doi:10.1186/s12929-015-0136-0)
Supplement: Additional file 1: — Sequence alignment of the MSP58 two NLSs across difference species, including human (h), mouse (m), Xenopus (x), zebrafish (z), quail (q) and Drosophila (d), and among members of the MSP58 family including p78 and MCRS2. [file 12929_2015_136_MOESM1_ESM.pdf]

Identification and characterization of nuclear and nucleolar localization signals in 58-kDa microspherule protein (MSP58)

Chuan-Pin Yang , Chi-Wu Chiang , Chang-Han Chen , Yi-Chao Lee , Mei-Hsiang Wu , Yi-Huan Tsou, Yu-San Yang, Wen-Chang Chang and Ding-Yen Lin

**p78**	**104**	**KRASSQAL-------------GTIPKRRSSSRFIKRKK**	**128**
**MCRS2**	**45**	**KRASSQAL-------------GTIPKRRSSSRFIKRKK**	**69**
**h MSP58**	**32**	**KRASSQAL-------------GTIPKRRSSSRFIKRKK**	**56**
**m MCRS1**	**32**	**KRASSQAL-------------GTIPKRRSSSRFIKRKK**	**56**
**x MSP58**	**27**	**KRSLPQGS -----------GLVPKRRSSSRFIKRKK**	**51**
**z MSP58**	**34**	**KRSAAQAFS------GAGLIPKRRSSSRSIKRKK**	**61**
**q TOJ3**	**102**	**KRGSVQPT ---------- GAVPKRRSSSRFIKRKK**	**126**
**d MSP58**	**70**	**STPIRNPISNLQIEQQNDQKRRSSSRTIKRKR**	**101**

**p78**	**185**	**PG--LTKRV KKSK**	**195**
**MCRS2**	**126**	**PG--LTKRV KKSK**	**136**
**h MSP58**	**113**	**PG--LTKRV KKSK**	**123**
**m MCRS1**	**113**	**PG--LTKRV KKSK**	**123**
**x MSP58**	**108**	**PS---IAKR I KKSK**	**118**
**z MSP58**	**118**	**PSSMTKRMKKNK**	**129**
**q TOJ3**	**180**	**PS--LAKRM KKSK**	**190**
**d MSP58**	**190**	**PLPVA PI V TAVA**	**201**

Additional file 1. Sequence alignment of the MSP58 two NLSs across difference species, including human (h), mouse (m), Xenopus (x), zebrafish (z), quail (q) and Drosophila (d), and among members of the MSP58 family including p78 and MCRS2.
